# Lymphoedema management knowledge and practices among patients attending filariasis morbidity control clinics in Gampaha District, Sri Lanka

**DOI:** 10.1186/1475-2883-3-6

**Published:** 2004-08-03

**Authors:** TGA Nilmini Chandrasena, Ranjan Premaratna, Nilanthi R de Silva

**Affiliations:** 1 Department of Parasitology, Faculty of Medicine, University of Kelaniya, PO Box 6 Talagolla Road, Ragama, Sri Lanka; 2 Department of Medicine, Faculty of Medicine, University of Kelaniya, PO Box 6 Talagolla Road, Ragama, Sri Lanka

## Abstract

**Background:**

Little information is available on methods of treatment practiced by patients affected by filarial lymphoedema in Sri Lanka. The frequency and duration of acute dematolymphangioadenitis (ADLA) attacks in these patients remain unclear. This study reports the knowledge, practices and perceptions regarding lymphoedema management and the burden of ADLA attacks among patients with lymphoedema.

**Methods:**

A semi-structured questionnaire was used to assess morbidity alleviation knowledge, practices and perceptions. The burden of ADLA attacks was assessed using one-year recall data.

**Results:**

66 patients (22 males, 44 females) with mean age 51.18 years (SD ± 13.9) were studied. Approximately two thirds of the patients were aware of the importance of skin and nail hygiene, limb elevation and use of footwear. Washing was practiced on a daily and twice daily basis by 40.9% and 48.5% respectively. However, limb elevation, exercise and use of footwear were practiced only by 21–42.4% (while seated and lying down), 6% and 34.8% respectively. The majority of patients considered regular intake of diethylcarbamazine citrate (DEC) important. Approximately two thirds (65.2%) had received health education from filariasis clinics. Among patients who sought private care (n = 48) the average cost of treatment for an ADLA attack was Rs. 737.91. Only 18.2% had feelings of isolation and reported community reactions ranging from sympathy to fear and ridicule.

**Conclusions:**

Filariasis morbidity control clinics play an essential role in the dissemination of morbidity control knowledge. Referral of lymphoedema patients to morbidity control clinics is recommended.

## Background

Lymphatic filariasis, identified as one of the leading causes of permanent disability worldwide, has been targeted for global elimination [1,2]. Interrupting transmission and controlling morbidity are the twin pillars of the global filariasis elimination programme [3]. Mass drug administration (MDA) for control of transmission began in the filarial endemic areas of Sri Lanka in 1999 and to-date 5-6 rounds of treatment have been completed. Morbidity management for those with filarial lymphoedema is still in its infancy. A regime of rigorous skin hygiene and simple self-help measures such as limb elevation, exercise, use of topical antibiotics and antifungals aimed at minimizing episodes of acute dermatolymphangioadenitis (ADLA) attacks and lymph stasis is the model recommended by the World Health Organization for management of filarial lymphoedema [4]. Little information is available on the methods of treatment practiced by patients affected by filarial lymphoedema in Sri Lanka. Furthermore, the frequency and the duration of debilitating acute attacks in Sri Lankan patients with filarial lymphoedema remains unclear. The objectives of this study were to determine the knowledge, practices and perceptions regarding lymphoedema management and the burden of ADLA attacks among patients with lymphoedema attending filariasis morbidity control clinics in an endemic area.

## Methods

The study was carried out in the Gampaha district in the Western province of Sri Lanka in November 2003. Ethical clearance for the study was obtained from the Ethics Committee, Faculty of Medicine, University of Kelaniya. Patients with lymphoedema attending morbidity control clinics conducted by the National Antifilariasis Campaign (AFC) were selected as the study population. These clinics are distributed in the operational areas of the AFC (Western, Northwestern and Southern provinces of Sri Lanka) and offer treatment exclusively to microfilaraemics and patients with chronic filariasis. Two weekly filariasis clinics (3.5 km apart) serve a population of 2,066,096 in a 1,387 km^2 ^area of the Gampaha district. A structured questionnaire was formulated to gather information on knowledge of lymphoedema management and limb care activities practiced along with frequency, treatment seeking behavior, cost and the degree of disability incurred in relation to ADLA attacks. A medical officer interviewed all patients regarding their disease history, examined and staged the limb affected by lymphoedema using World Health Organization criteria [5]. The presence of entry lesions, state of skin and nail hygiene of the affected part were recorded. Informed consent was obtained from the study participants prior to administering the questionnaire. The pre-tested questionnaire was administered in the local language: each patient was interviewed in depth by medical and paramedical staff (trained in lymphatic filariasis disability management) to explore their knowledge of lymphoedema management and the limb care activities practiced. The questions on lymphoedema management knowledge focused on the degree of importance attached to limb washing, elevation, exercise, nail hygiene, minimizing skin trauma, use of foot wear, intake of diethylcarbamazine citrate (DEC) and use of antiseptics/ topical antibiotics on entry lesions. The limb care activities practiced by the subjects were assessed by exploring how frequently they practiced the activities mentioned above in the knowledge component of the questionnaire. Questions were asked on prevalence of acute attacks during the preceding year, treatment seeking behavior during an acute attack, treatment cost per acute attack and the degree of disability incurred by an acute attack. An episode of ADLA was defined by the following criteria; painful swelling of the affected part with increased local warmth, redness and tenderness with or without associated constitutional symptoms such as fever, nausea and vomiting.

Patient perceptions of disease reversibility and community attitudes towards their altered physical appearance were also explored. At the end of the interview all patients were educated on lymphoedema management practices recommended by the World Health Organization [5].

## Results

A total of 66 patients [male:female ratio 22 (43.3%): 44 (66.7%)] with a mean age of 51.18 years (SD ± 13.9) were enrolled in the study. A substantial proportion of this study sample (24.3%) had received only primary school level education.

Most subjects had lymphoedema of a single lower limb [n = 56, 84.8%]. Both lower limbs were affected in 6 (9%), a single upper limb in 3 (4.4%) and a single upper limb and a breast in one subject. The oedema was completely reversible (stage 1) in 21 (31.8%) subjects; 30 (45.5%) were in stage 2. Subjects with non-reversible lymphoedema had associated shallow skin folds 11 (16.7%) (stage 3), one (1.5%) had skin knobs (stage 4) and three (4.5%) had deep skin folds (stage 5). The common entry lesions were sole fissures (n = 14), minor injuries (n = 5) and eczema (n = 2).

### Knowledge

**Figure 1:**
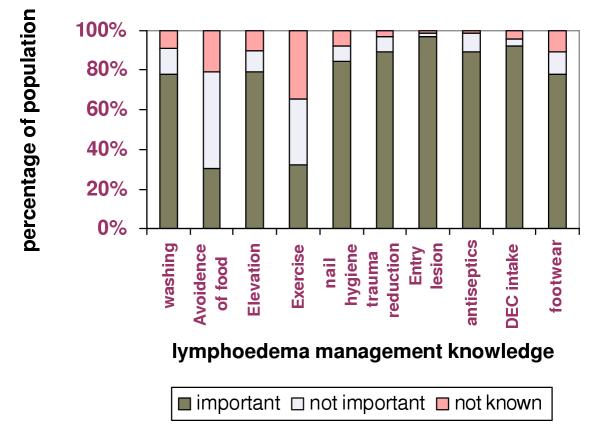
**Lymphoedema management knowledge of the population. **Washing: Washing the affected part with soap and water Avoidance of foods: Avoidance of food items Elevation: Keeping the affected limb elevated Exercise: Exercising the affected limb Nail hygiene: Keeping the nails of the affected limb clean and short Trauma reduction: Minimizing trauma to the affected part Entry lesion survey: Examining the affected part for entry lesions Antiseptics: Use of antiseptics on entry lesions on the affected part Footwear: Use of footwear for lower limb lymphoedema

Knowledge and attitudes regarding lymphoedema management are summarized in Figure 1. Almost two thirds of the population were aware of the importance of skin and nail hygiene, limb elevation and use of foot wear. However, exercising the affected part was considered important by only 21 (31.8%) subjects. Regular treatment with DEC and avoidance of certain food items (fatty foods) were considered as important measures by 92% and 30.3% of the subjects respectively. Most patients (71.2%) had received advice regarding lymphoedema management from the medical officer at the morbidity control clinic (65.2%), from general practitioners (7.6%) and public health inspectors (6.7%). Few had also acquired their knowledge by reading leaflets / booklets on lymphatic filariasis (18.2%).

### Practices

Most of the study subjects washed the affected body part with soap and water either daily (n = 29, 40.9%) or twice daily (n = 32, 48.5%). A significant proportion of the study sample (n = 19, 29%) also used coir (coconut fibre) to rub the skin while washing. Half and one third of the study population practiced limb elevation on a regular basis while lying and sitting down respectively. Exercising the affected limb was practiced on a regular basis only by a minority (n = 4, 6%) of the patients. Footwear was used on a regular basis by 34.8% of the subjects, while 48.5% used them only outdoors. 7.6% of the sample did not use footwear at all.

Almost all the subjects took DEC daily (n = 59, 89.4%) or periodically (n = 7, 10.6%). Only 14.3% required assistance to clean the affected body part and it was usually a family member who assisted them. The need for assistance was associated with advanced age and/or stage of lymphoedema and ADLA attacks. Assistance was required not only for cleaning the affected part (fetching water, washing) but also for surveying for entry lesions owing to failing sight in the elderly.

### ADLA attacks

Thirty-one (46.97%) subjects had one or more ADLA attacks during the preceding year. Although around 50% of those who reported acute attacks suffered only one, some had experienced as many as 12 attacks during the past year (Table 1). The mean number of ADLA attacks tended to rise with stage of lymphoedema.

**Table 1:** Frequency of ADLA attacks according to the stage of lymphoedema

ADLA attacks/year>	Numbers affected according to lymphoedema stage						
	
	Stage 1 n = 21	Stage 2 n = 30	Stage 3 n = 11	Stage 4 n = 1	Stage 5 n = 3	Stage 6 n = 0	Stage 7 n = 0
1	7	6	2	0	1	0	0
2	0	1	0	0	0	0	0
3	2	3	0	0	0	0	0
4	1	2	0	0	1	0	0
5	0	0	1	0	0	0	0
6	0	0	2	0	0	0	0
12	0	0	1	0	1	0	0
Mean	0.81	0.83	2.82	0	5.67	-	-

Out patient departments (OPD) of Government hospitals (44.9%), private practitioners (30.6%) and filariasis clinics (14.3%) were the preferred sources of treatment for acute attacks. The treatment cost of an acute attack ranged from Rs.100 to 3500 (1 US$ = 100 SLR) with an average cost of Rs. 737.91 per attack for those who sought private care (n = 48). The average duration of an acute attack was 3.5 days. Fifty two percent of those who had experienced an acute attack in the past (n = 48) were totally incapacitated (unable to attend to any domestic / economic activity) while 31.3% were moderately incapacitated (able to attend to some domestic / economic activity) for the duration of the acute attack.

Almost all subjects (n = 60, 92.3%) believed that their lymphoedema was reversible and treatment with antiparasitic drugs was identified as the most important therapeutic option for reversing/ halting the progression of the disease.

Only 18.2% of the study sample felt that they were being shunned by the society and that their altered physical appearance elicited various reactions ranging from sympathy to fear and ridicule from the community.

### Non-clinic attendees

During the period of this study an attempt was also made to collect comparable data from non-clinic attendees. However, it was possible to identify only 10 subjects, all seeking treatment in the OPD of the University Teaching Hospital. These 10 patients were in lymphoedema stages I (n = 2), II (n = 2) and III (n = 6) respectively. According to the data elicited by using the same questionnaire, 8 of these 10 non-clinic attendees (80%) were unaware of the currently recommended disability management measures. Further analysis was not carried out since the sample size was not comparable with that of clinic attendees.

## Discussion

The results of this survey indicate that the majority of patients attending filariasis clinics are aware of the importance of the currently recommended morbidity control measures. It was also encouraging to note that many practiced at least some of the morbidity control measures that they had learnt, especially washing the affected part with soap and clean water once or twice a day. Although many were aware of the importance of minimizing skin trauma, a sizeable proportion used coir to clean the skin of the affected part during washing. This is a harmful practice which could traumatize the skin and lead to entry lesions; therefore demonstration of suitable alternatives is clearly necessary. A community educational programme may be especially beneficial as it would also involve the family members of the affected population who are often the caregivers of patients affected with severe grades of lymphoedema. Involving the family in the care of the patient will also help to reduce the feelings of stigma, isolation and neglect experienced by some of the patients.

Limb elevation was identified as an important measure by most patients but not practiced on a regular basis by many. Poor compliance with limb elevation has also been reported in Southern India [6]. The authors attributed this to inconvenience and the belief that it caused only a temporary reduction in lymphoedema.

Movement and elevation has been shown to play an important role on the lymphatic and venous systems of the body [7-9]. However, only 31.8% of the study population was aware of the importance of exercise and very few practiced it on a regular basis. Patients with lymphoedema tend to become increasingly immobile and the affected limb is most often in a dependent position causing venous hypertension and resultant overloading of the failing lymphatics [9]. Therefore, health education emphasizing the long-term benefits of exercise and limb elevation should be provided in a comprehensive manner to patients and caregivers. It is also important to ensure that footwear is used indoors as well as outdoors, as the commonest entry lesions noted in the study population were sole fissures which are highly likely to become infected when walking barefoot. In contrast to previous reports [10], fungal infections of the interdigital spaces were not seen in any participants in the present study – perhaps because most of the study population had early grades of lymphoedema.

The practice of prescribing DEC on a regular basis to patients affected with chronic disease needs to be discouraged as there is no conclusive evidence regarding any beneficial effects of long term use of DEC in the management of lymphoedema [10,11]. Misconceptions regarding the need to avoid fatty foods seem to be based on the belief that such foods may increase the volume (fatness) of the affected limb. Such beliefs may have originated from health messages of the lymphatic filariasis program with regard to management of chyluria. These misconceptions need to be corrected, as patients tend to avoid some commonly available food items.

Although the data regarding acute attacks may not be highly accurate as it was based on one-year recall, there is reason to believe that recall data reliably reflect the burden imposed by ADLA attacks on the affected population [12,13].

Where patients had sought private care for ADLA attacks, average total expenditure on treatment was about Rs. 737.91 per attack – it is not surprising, therefore, that more than 50% of the population (59.1%) sought treatment from Government health facilities which provide free health care. Income lost by patients during an ADLA attack due to physical incapacitation was not determined in this study.

The findings of this study may not be generalized to all patients with lymphoedema in the Gampaha district as the subjects surveyed were patients already enrolled in morbidity control clinics. Limited information gathered from non-clinic attendees indicated that the level of knowledge among clinic attendees might be far superior to that of non-clinic attendees.

Lymphatic filariasis continues to be a major public health burden. Globally an estimated 15 million suffer disfiguring symptoms of lymphoedema and elephantiasis [14]. Although the national burden of lymphoedema has not been fully quantified, a prevalence of 3% has been reported from some parts of the country [15]. Therefore, in Sri Lanka, morbidity management needs to be strengthened.

## Conclusions

Referral of lymphoedema patients to morbidity control clinics is recommended as they appear to play an important role in the dissemination of morbidity control knowledge. The services of these clinics needs to be further improved to ensure that all clinic attendees receive proper education on disability management. Training and education of specialists in the management of lymphoedema may be a useful investment for eliminating the handicap caused by chronic lymphatic filariasis.

## List of abbreviations used

ADLA: Acute dematolymphangioadenitis

MDA: Mass drug administration programme

AFC: Anti Filariasis Campaign

DEC: Diethylcarbamazine citrate

SD: Standard deviation

OPD: Out patients Department

SLR: Sri Lanka rupee

US$: U.S. dollar

## Competing interests

None declared

## Authors Contributions

TC designed the questionnaire, conducted the survey and drafted the manuscript. RP Participated in data collection and interpretation. NS provided overall supervision of the study and preparation of the manuscript. All authors read and approved the final manuscript.
